# LoCo: a novel main chain scoring function for protein structure prediction based on local coordinates

**DOI:** 10.1186/1471-2105-12-368

**Published:** 2011-09-15

**Authors:** Stewart E Moughon, Ram Samudrala

**Affiliations:** 1 Department of Microbiology, University of Washington, Box 357735, Seattle, Washington, 98195-7242, USA

## Abstract

**Background:**

Successful protein structure prediction requires accurate low-resolution scoring functions so that protein main chain conformations that are close to the native can be identified. Once that is accomplished, a more detailed and time-consuming treatment to produce all-atom models can be undertaken. The earliest low-resolution scoring used simple distance-based "contact potentials," but more recently, the relative orientations of interacting amino acids have been taken into account to improve performance.

**Results:**

We developed a new knowledge-based scoring function, LoCo, that locates the interaction partners of each individual residue within a local coordinate system based only on the position of its main chain N, C_α _and C atoms. LoCo was trained on a large set of experimentally determined structures and optimized using standard sets of modeled structures, or "decoys." No structure used to train or optimize the function was included among those used to test it. When tested against 29 other published main chain functions on a group of 77 commonly used decoy sets, our function outperformed all others in C_α _RMSD rank of the best-scoring decoy, with statistically significant p-values < 0.05 for 26 out of the 29 other functions considered. LoCo is fast, requiring on average less than 6 microseconds per residue for interaction and scoring on commonly-used computer hardware.

**Conclusions:**

Our function demonstrates an unmatched combination of accuracy, speed, and simplicity and shows excellent promise for protein structure prediction. Broader applications may include protein-protein interactions and protein design.

## Background

Protein structure prediction is a difficult problem for several reasons. The forces that determine structure are not fully understood, at least quantitatively [1]. While there is a good qualitative understanding of these forces, there is still no accurate way to calculate the free energy gained by the burial of hydrophobic atoms away from solvent. Nor can we accurately model the highly variable dielectric constant in the interior of a protein. In addition, to correctly predict the conformation of a protein we must first represent it in a computer, and any computational representation of that protein must significantly simplify its components and their interactions. Any change to one part of a protein we are trying to model may affect many other parts of that model.

The first descriptions of protein structure at atomic detail were given by Pauling, Corey and Branson in 1951 [2-10]. Only secondary structures were described, however, and not all of them have been observed in nature. Nevertheless, it was an extraordinary achievement. The first three-dimensional protein fold described was the structure of myoglobin, solved by Kendrew, et al., in 1958 [11].

Difficulties using these descriptions to predict protein structure soon became apparent. In the late 1960s, it was noted that the number of possible conformations of a typical polypeptide chain is so large that it must have a pathway in the course of protein folding, the so-called "Levinthal Paradox [12,13]."

Protein structure can be viewed in a hierarchical manner, where oligomers are made up of polypeptide chains, which are made up of amino acids, which are made up of atoms. It may be considered conceptually to be determined hierarchically as well, with primary structure (the sequence) determining secondary and tertiary structure. Since each possible main chain conformation can have an astronomically large number of possible combinations of amino acid side chain arrangements, one approach to tackle the problem in a hierarchical way is by modeling a manageable number of the most likely main chain conformations before addressing the problem of amino acid side chains. In the initial stages, coarse-grained functions using highly simplified representations of amino acids are employed to quickly evaluate a large number of proposed main chain conformations [14,15]. Only a small fraction of these structures are selected for more detailed assessment. If the low-resolution functions used to select that small fraction are unable to discriminate near-native main chains from incorrect ones, then a successful prediction is effectively impossible using this approach.

It is therefore necessary to sample all possible main chain conformations in such a way as to ensure that near-native structures will be among those evaluated. As a practical consideration, it is also important to model the smallest number of main chain conformations needed to ensure that conformations good enough to be considered successful predictions (or able to lead to successful predictions) are among those sampled. Just as important, one must be able to evaluate the sampled conformations in reasonable computing time.

In this work, we address the problem of rapid and accurate evaluation of sampled conformations. To do this we use sets of "decoys"--non- and near-native conformations of a given protein sequence that have been proposed in the course of protein structure prediction or generated by making alterations to a native structure. The goal is to be able to discriminate the native and near-native conformations from the non-native ones. Further, we focus on the problem of quickly and accurately assessing proposed main chain conformations, ignoring side chains.

### Types of functions

There are two categories of functions that are applied to protein structures to evaluate their likelihood of being correct: physics-based functions [16] and knowledge-based functions [17]. Physics-based functions attempt to model the actual physics that determine the behavior of proteins. Knowledge-based functions are derived from statistical profiles taken from sets of known protein structures. To create these profiles, some representation of a protein or its constituent parts is chosen, then the known structures in the set are categorized according to the chosen representation. Functions derived from this profile allow any protein conformation to be evaluated according to how closely it corresponds to the profile.

When examining main chains only, no individual amino acid can be considered to be in any particular side chain conformation. Since this undetermined state does not correspond to any physical entity, knowledge-based functions must be used to evaluate it. These functions take a number of forms. One common approach is to measure the separations between all pairs of residues and apply the function to all of them that fall below a given cutoff distance [18-38]. These separations are typically between C_α _atoms, C_β _atoms or presumed centers of mass for each residue. These so-called "contact" potentials depend on the identities of both residues. They typically make use of a pairwise matrix of interaction values that may or may not be adjusted for the distance between residues.

Since the early development of coarse-grained contact potentials, progress has been steady. While the interaction representations have remained similar, the discrimination power of the matrices has been improved. Some innovations have included quasi-chemical treatments [24,29,32], hydrophobic energies [21,29,39] and more sophisticated statistical treatments [28,33]. Still, even developers of these potentials have acknowledged their insufficiency for protein structure prediction by themselves [30,35]. More recent work has demonstrated further difficulties with statistical potentials based on preferential interactions [40,41].

Amino acid interaction potentials have begun to include the relative orientations of pairs of residues as well. Buchete, Straub and Thirumalai calculated a five-dimensional potential with a local coordinate system generated around the main chain C_α _and side chain C_β _and C_γ _atoms [42]. Mukherjeee, Bhimalapuram and Bagchi developed their potential around a single ellipsoidal representation of the side chain [43]. Makino and Itoh achieved excellent discrimination of native structures from decoys with a six-term orientation-dependent potential [44]. Rykunov and Fiser made use of a "shuffled reference state" to improve the performance of their orientation-dependent potential [45].

We continue this trend of using additional geometric information in the consideration of residue-residue interactions and present a new coarse-grained function for evaluating protein main chain conformations by scoring interactions between amino acids within a single polypeptide chain, using only the positions of main chain N, C_α _and C atoms. All pairwise residue-residue interactions are actually considered to be two interactions: one from the perspective of each residue. All other residues within a specified cutoff distance are considered to be potential interaction partners, although we do exclude from scoring some number of immediate neighbors in the chain. We use a large pre-calculated database of interaction potentials for quick scoring.

Scoring is carried out by locating all interaction partners for any given residue within a local Cartesian coordinate system defined by that residue's main chain N, C_α _and C atoms. This local coordinate system is divided into cubic 1Å bins, and every interaction partner is assigned to a bin. The score for any interaction is based on the likelihood of observing a particular residue at those locally-defined coordinates, given the type of the residue for which the coordinate system is constructed and the type of the interaction partner observed. This scoring function we have named *LoCo *(for **Lo**cal **Co**ordinates). It yields state of the art performance with a speed and simplicity that is unmatched by any other function at its level.

## Methods

### Overview

The fundamental idea behind LoCo scoring is that characteristic shapes of amino acids lead to characteristic geometric relationships between interacting residues in a native structure. The interior of a properly folded protein is tightly packed. Main chain atoms typically form a rigid planar structure between C_α _atoms, and steric considerations generally confine the side chain atom positions into one of a number of rotamers. These restrictions on the overall shapes that amino acids generally indicate that there are a limited number of ways they will typically fit well together, both spatially and energetically.

The relationships we exploit are relative positions in three-dimensional space. Most coarse-grained potentials have relied simply on distances between C_α _atoms, C_β _atoms or centers of mass [35]. By using additional dimensions to characterize residue-residue interactions, our method is more specific about which interactions are favorable and which are not. Since it has more dimensions, it requires a considerably larger and more detailed set of parameter tables than have generally been used, which is not a limitation it once was due to ever-increasing storage and memory.

### LoCo Methodology

**Figure 1:**
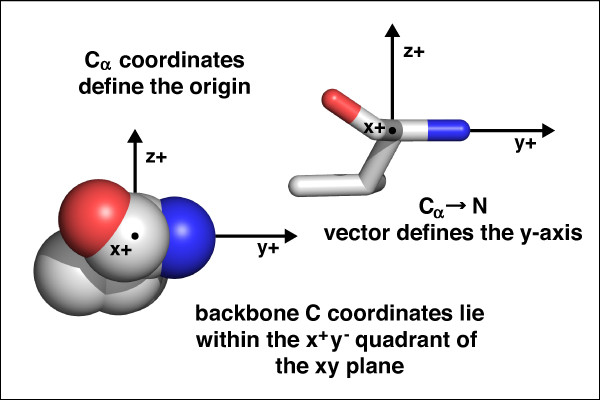
**The "LoCo" local coordinate system**. Shown is a single valine residue and its local coordinate system, seen looking down the positive x-axis. The center of the C_α _atom is defined to be the origin. The positive y-axis passes through the main chain N coordinates. The positive X axis is placed such that the main chain C coordinates fall within the xy plane in the positive X direction. The coordinate system is right-handed. Both stick and sphere representations are presented for clarity.

LoCo scoring takes place within a local coordinate system defined by the main chain N, C_α _and C atoms of the residue being scored (Figure 1) for any given amino acid. The C_α _is at the origin of this coordinate system. The N coordinates define the y-axis, and position of the main chain C atom defines the x- and z-axes. A different coordinate system is generated for each residue. We refer to the amino acid at the origin as the "observing" residue and all nearby residues eligible to interact with the observing residue as "partner" residues.

**Figure 2:**
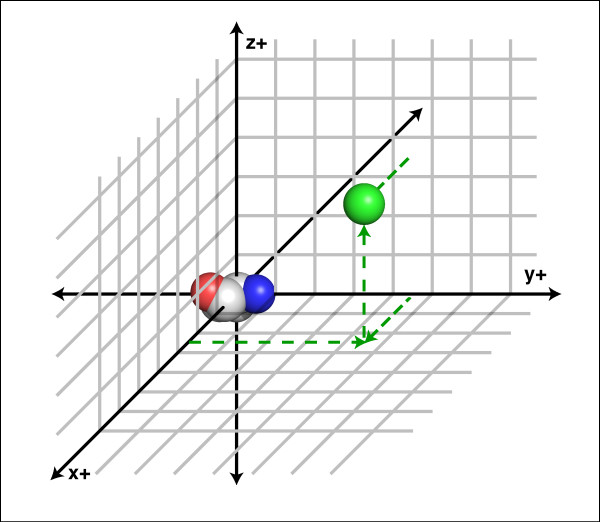
**A single LoCo interaction**. An interaction within a LoCo coordinate system is illustrated. Bins shown are approximately 3× actual size for clarity. Bin boundaries are counted from the origin. A single valine is placed as shown in Figure 1. The C_α _atom of an interacting residue is displayed separately in green within bin +3, +5, +4.

To score an interaction using LoCo, the C_α _atom of each partner residue is located within a particular 1Å cubic bin of the coordinate system of the observing residue (Figure 2). The partner residue is then assigned a score based on the likelihood of its being observed in that bin, given the types of both residues. The total score for any given observing residue is the sum of all the scores for its partners, and the score for the protein is the sum of all residue scores when every residue has been treated as an observing residue.

The individual interaction scores are derived from statistics that have been obtained from a large set of non-homologous protein domains. Here is the formula:

$$\text{S = }\overset{N_{1}}{\underset{i=1}{\sum }}\overset{N_{2}}{\underset{j=1}{\sum }}\text{ - ln}\left(\frac{j_{obs}\left(xyz\right)}{j_{\exp}\left(xyz\right)}\right)$$

where S is the total score for all pairwise residue-residue interactions, and *i *and *j *are the observing and interacting residues, respectively. This is equivalent to the Boltzmann equation [46], where the quantity *j_obs_*(*xyz*) is the number of times a residue of type *i *has observed a residue of type *j *in the training set at bin coordinates *x*, *y *and *z *in its local coordinate system. The reference state, *j_exp_*(*xyz*) is the number of times a residue of type *j *would be expected to be observed at those coordinates. *N*_1 _represents all amino acids in the polypeptide chain; *N*_2 _represents only those residues that are eligible to be interaction partners for *i*.

We define the reference state *j_exp_*(*xyz*) to be the mean number of observations of all residue types at bin coordinates *xyz*, which is the total number of observations at those coordinates for any residue types *i *and *j *divided by the total number of possible *ij *combinations, totaling 400 (since there are 20 amino acid types). This mean includes zero-count cases. Since we cannot take the logarithm of zero, bins with no observed counts are assigned a penalty equal to a some multiple of the worst-scoring bin for any observed *ij *interaction.

The value of this zero-count penalty affects the accuracy of scoring, as do eligibility criteria for partner residues. Varying the cutoff distance for eligible partners affects performance. Since the C_α_- C_α _separations across peptide bonds are effectively fixed and the number of well-populated Φ and Ψ angles fairly restricted, we did not score immediate sequence neighbors of the observing residue.

The values of these three parameters--the interaction cutoff distance, the number of neighboring residues to exclude from scoring and the size of the zero-count penalty--were chosen using a training group of decoy structures before the final version was evaluated on an independent group of decoy sets.

### Function Training

Training of the LoCo function took place in two separate stages: generation of the scoring database and optimization of its parameters. Each database was generated by assigning a probability-based score to every possible state of the system using a large set of known protein structures that are held to be representative of correct structures. We presume that this set, although not complete, captures enough information about residue-residue interactions to be of predictive value. Parameter optimization involved finding the version of the function with the best-performing set of values (from among those tested) for the three interaction parameters described above.

All observed counts for the generation of all versions of the LoCo scoring databases were taken from the ASTRAL 1.73 set [47] of 9527 non-homologous protein domains. As noted above, we also used a "training group" of 154 decoy sets to find an optimal set of function parameters. All structures in both the training group and the testing group that were part of the ASTRAL set were removed before the potentials used to score each group were generated.

When optimizing our function parameters, we followed a process of tenfold cross-validation to ensure that even within the training group no function was evaluated on a group of decoy sets that had been used to select it. Interatomic cutoff distances of 8Å -20Å were tested in 2Å increments. From 1 to 4 chain neighbors on both the N- and C-terminal sides of the observing residue were not scored. We established a baseline zero-count penalty equal to the worst score calculated for each pair of residue types, then tested penalties equal to 1, 2 or 3 times the baseline. This gave us a total of 84 different versions of the LoCo scoring function.

The training group was divided into ten randomly selected subsets--six containing 15 decoy sets and four containing 16. Ten different groupings of nine of these subsets were scored using all 84 versions of the LoCo function, and the average C_α _RMSD between the native and the best-scoring non-native structure was calculated for each version. The version of LoCo with the lowest average C_α _RMSD across all nine subsets was used to score the remaining subset. The LoCo version selected was the one with the lowest overall average C_α _RMSD among all ten remaining subsets.

This tenfold cross-validation procedure was carried out ten separate times to ensure that the outcome was not dependent on a particular random selection of the subsets. In every case the best performance was achieved with a cutoff distance of 14Å, with only a single residue on either side of each residue excluded from scoring and with a zero-count penalty equal to 3 times the worst observed score for each particular combination of amino acid types. This version of the LoCo scoring database was used for our final performance testing.

### Decoy sets for evaluation of scoring functions

The purpose of protein main chain scoring functions is to discriminate near-native from non-native conformations. "Decoy" structures representing a mix of near- and non-native conformations for a particular amino acid sequence, commonly generated in the course of protein structure prediction, are often used to evaluate them. Such sets typically include the native structure.

We decided to follow the model of Makino and Itoh [44], to optimize parameters before we could test the scoring performance of LoCo. We used the same 231 decoy sets from the "Decoys R Us" database http://dd.compbio.washington.edu/[48], the 62-protein "Rosetta" set from David Baker's group http://depts.washington.edu/bakerpg/decoys/rosetta_decoys_62proteins.tgz, and the "moulder" set ftp://salilab.org/decoys/[49,50] from Andrej Sali's group. These are among the most widely used decoy sets in the field. We divided these 231 decoy sets into the same two groups, a 154 set group for function optimization (the "training group") and a 77 set group for performance evaluation (the "testing group").

Since we are pursuing main chain structure discrimination only, all side chain atoms except C_β_s were removed from the decoys. Although C_β _atoms are not part of the main chain, their positions do not change (at least ideally) as side chain conformations do, so they can be included in an initial search for main chain conformations. C_β _atoms are not used in LoCo scoring, but are used by some of the other functions in our comparisons.

### Function comparisons

Performance of the LoCo potential was tested against a total of 29 other published functions for main chain evaluation. Twenty-six of these functions are from the Jernigan Lab's Knowledge-based Potential Server: http://gor.bb.iastate.edu/potential/, representing some of the widely used contact potentials of the last 30 years. Also among the 26 are 3 more recently-developed functions from the Jernigan Group--the Four-body and General-four-body [38], and the Short-range [27]--that are not simple contact potentials.

The remaining 23 contact potentials are identified here with the same codes used on the Jernigan server: Qa, Qm, Qp [37], HLPL, MJPL [25], SKOa, SKOb, SJKG [29,34], MJ1, MJ2h, MJ3, MJ3h [20,24,32], TS [18], BT [31], BFKV [36], TD [26], TEl, TEs [35], RO [19], MS [23], GKS [22], VD [30], BL [21], and MSBM [28,33].

Three more modern potentials are considered as well. The program ProSa 2003 is from the group of Manfred Sippl [46,51,52] and is available from the Center of Applied Molecular Engineering: http://www.came.sbg.ac.at/. Two recently developed functions that explicitly take the relative orientations of interacting residues into account are DFMAC, by Makino and Itoh [44], and RF_CB_SRS_OD, by Rykunov and Fiser [45]. Executables of both are available from their authors.

The functions from the Jernigan Group server encompass a wide variety of approaches: the oldest (TS) was published in 1976, and the newest (the Four-body and General-four-body) in 2007. Some are simple contact potentials that assign a score to all pairs or residues found within a given cutoff distance of one another. Other functions in the set assign distance-dependent scores to pairs within the cutoff distance. Not all functions are purely knowledge-based: several use techniques such as quasi-chemical approximation or attempt to calculate hydrophobic energies. Some of the publications represented note the insufficiency of contact potentials alone for protein structure prediction.

ProSa 2003 generates three scores for every residue: a pair score, a surface score and a combined score. Scores used for comparison are the sum of all individual residue combined scores, which outperformed both the individual pair and surface terms. The potentials used were the "prosa2003.pair-cb" and "prosa2003.surf-cb" included with the distribution.

The DFMAC function is a linear combination of six separate weighted pseudo-energy potentials involving pairwise C_α _separations, relative orientations of pseudo C_α_→ C_β _vectors, main chain-to-main chain pseudo-hydrogen bonding, Φ/Ψ angle pairings between residues, individual residue ω angles, and the number of other C_α _atoms surrounding each C_α _atom. These six potentials have sixteen independent parameters that were "tuned" on the same group of 154 decoy sets that we used for our parameter training. Once the most favorable set of those sixteen parameters was selected for that training group, the weights of all six components of the function were similarly optimized before the function was tested on the same 77 decoy set testing group we have used here.

The RF_CB_SRS_OD function groups residue-residue interactions into three categories: residues facing in the same direction, residue facing toward each other and residues facing away from each other. "Facing" in this context refers to the direction of each amino acid's C_α_→ C_β _vector. A "shuffled" reference state is created by randomizing the sequence position of all residues in the protein.

### Performance Measures

We use five performance measures for native structure recognition: Rank_nat_, RMSD_best_, Z_nat_, CC_nat _and FE_nat_. Eight measures--R_B1_, R_B10_, RMSD_decoy_, Z_decoy_, CC_decoy_, FE_decoy_, log(P_B1_) and log(P_B10_)--are used for decoy discrimination. Rank_nat _is the score rank of the native structure among all decoys. RMSD_best _is the C_α _RMSD of the best-scoring structure, including the native. Z_nat _is the Z-score of the score of the native structure relative to all other scores (native included) in that decoy set. CC_nat _is the Pearson's correlation coefficient between score and C_α _RMSD for all structures in the set, including the native. FE_nat _is the fraction enrichment among all decoys (native included) after scoring. The fraction enrichment is defined as the fraction of the top 10% of our structures by C_α _RMSD that are found among the top 10% by score. We express the fraction enrichment as a percentage for clarity.

R_B1 _is the C_α _RMSD ranking among decoys only (native excluded) of our best-scoring structure. R_B10 _is the lowest C_α _RMSD rank among the 10 best-scoring structures from the decoy set (not including the native). RMSD_decoy _is the Cα RMSD of the best-scoring structure, excluding the native. Z_decoy _is the Z-score of the score of the lowest-RMSD decoy relative to all other scores (not including the native) in that decoy set. CC_decoy _is the correlation coefficient between score and C_α _RMSD for all structures in the set, excluding the native. FE_decoy _is the fraction enrichment among all decoys (native excluded) after scoring. The measures log(P_B1_) and log(P_B10_) are the common logarithms of the probabilities of selecting the R_B1 _and R_B10 _structures. These probabilities are simply the values of R_B1 _and R_B10 _divided by the total number of decoy structures in the set (excluding the native).

## Results

### Native recognition vs. decoy discrimination

The performance measures we use fall into two categories: native recognition and decoy discrimination. Native recognition is the ability to recognize the native structure from among all decoys in the set. Decoy discrimination is the ability to pick out one or more near-native structures within the set. A good scoring function should be able to pick out the native, at a minimum. However, the likelihood of reproducing a completely correct structure in the course of sampling different conformations is quite low. For practical use, a good scoring function must be able to distinguish near-natives from non-native structures.

### Training and testing group comparison

We used separate groups of decoy sets for optimizing the variable parameters of LoCo and for testing its performance against other functions. A comparison of LoCo scores achieved with training and testing groups is in Tables 1 and 2. Table 1 shows the differences between these groups in native structure recognition. Table 2 shows these differences for decoy discrimination. Roughly comparable results were achieved with both groups, though the test group did yield somewhat better results across the board.

**Table 1:** Training vs. testing groups: native recognition test

	Total sets	# of Natives	# RMSD < 2Å	# RMSD < 5Å	Rank_nat_	C_α _RMSD_best_	Z_nat_	CC_nat_	FE_nat _(%)
Training group	154	57	112	136	47.1	2.10	1.587	0.478	32.9
Testing group	77	38	60	70	13.4	1.62	1.805	0.519	36.6

Comparison of LoCo performance for native structure recognition on both the training and testing groups of decoy sets is shown. Results are roughly comparable, though the testing group did somewhat better across the board. Natives, RMSD < 2Å and RMSD < 5Å refer to the number of times the best-scoring structure in a particular group is either the native structure (0Å C_α _RMSD) or within 2Å or 5Å C_α _RMSD from the native structure, respectively. All other measures are averages over every decoy set in the group. Definitions of measures used are provided in the Performance Measures subsection at the end of **Methods**. In summary, lower scores are better for Rank_nat _and RMSD_best_; higher scores are better for all the other measures..

**Table 2:** Training vs. testing groups: decoy discrimination test

	Total sets	R_B1_	R_B10_	RMSD_decoy_	Z_decoy_	CC_decoy_	FE_decoy_	log(P_B1_)	log(P_B10_)
Training group	154	172.0	39.2	3.75	0.829	0.461	29.9	-0.773	-1.491
Testing group	77	154.8	5.6	3.51	0.938	0.505	31.4	-0.864	-1.640

Comparison of LoCo performance for decoy discrimination on both the training and testing groups of decoy sets is shown. Again, results are roughly comparable, though the testing group did somewhat better overall. All measures are averages over every decoy set in the group. Definitions of measures used are provided in the Performance Measures subsection at the end of **Methods**. In summary, lower scores are better for R_B1_, R_B10_, RMSD_decoy_, log(P_B1_) and log(P_B10_); higher scores are better for Z_decoy_, CC_decoy _and FE_decoy_.

We consider decoys that are less than 5Å C_α _RMSD from the native to be "near native" structures and decoys that are less than 2Å to be "very near native." We include the numbers of near native and very near native structures found with our "native recognition" measure in Table 1. For the training group, the best-scoring structure in each set was very near native for 112 of 154 decoy sets (72.7% of the time) and near native for 136 sets (88.3% of the time). For the test group, the best-scoring structures were very near native in 60 of 77 cases (77.9%) and near native for 70 of 77 sets (90.9% of the time). The average C_α _RMSD (from the native) of the best-scoring structures from all of the training group was 2.10Å. For the test group it was 1.62Å, a difference of less than 0.5Å. All performance measures, with the exception of numbers of near native and very near native structures, are explained in *Performance measures *at the end of **Methods**.

Differences between training and testing groups were smaller for decoy discrimination. The difference between the average C_α _RMSD (from the native) for the best-scoring non-native structure was less than 0.25Å between the groups. It is perhaps not surprising that these measures were so close, since that was the metric for which the training group was optimized. Again, test group measures were somewhat better but not largely so, with the exception of R_B10_, indicating that LoCo was significantly more able to place one of the ten nearest-native structures among its ten top-scoring decoys.

### Main chain function performance

Native recognition performance is demonstrated in Table 3. The performance of the top four functions, LoCo, DFMAC, RF_CB_SRS_OD and ProSa 2003, is superior to that of the remaining potentials. LoCo outperforms every function except DFMAC. However, the relatively larger differences between LoCo and DFMAC in Rank_nat _and Z_nat _may partly be due to the inclusion of an ω-angle component in DFMAC, which is of limited practical utility (see *Omega angles*, in **Discussion**).

**Table 3:** Function comparison: native recognition

	Rank_nat_	RMSD_best_	Z_nat_	CC_nat_	FE_nat _(%)
**LoCo**	13.4	1.62	1.805	0.519	36.6
**DFMAC**	6.7	1.17	2.630	0.562	38.3
**RF_CB_SRS_OD**	19.3	2.68	1.508	0.464	31.3
**ProSa 2003**	44.0	2.39	1.288	0.491	33.8
**Four-body**	81.8	4.87	0.621	0.334	20.4
**General-four-body**	56.3	4.67	0.797	0.311	18.9
**Short-range**	87.5	4.87	0.353	0.257	13.0
**BFKV**	54.5	3.54	0.774	0.397	24.7
**BT**	45.8	3.85	0.744	0.390	23.2
**GKS**	28.5	5.42	0.229	0.235	12.3
**HLPL**	31.4	3.37	0.602	0.383	24.8
**MJ1**	124.5	3.79	0.014	0.336	20.4
**MJ2h**	101.3	3.20	0.324	0.377	23.0
**MJ3**	50.6	4.69	0.401	0.244	15.7
**MJ3h**	52.1	3.63	0.733	0.410	26.3
**MJPL**	57.8	3.33	0.246	0.353	23.0
**MS**	54.0	4.94	0.419	0.234	13.3
**MSBM**	54.2	5.77	0.119	0.159	7.5
**Qa**	37.4	4.72	0.749	0.296	20.4
**Qm**	31.6	4.35	0.723	0.275	19.2
**Qp**	28.8	3.12	0.513	0.365	24.3
**RO**	248.3	5.67	0.287	0.248	17.6
**SKJG**	34.1	4.16	0.756	0.369	21.4
**SKOa**	33.1	4.35	0.664	0.352	20.5
**SKOb**	30.3	4.11	0.652	0.363	21.8
**TD**	47.7	3.81	0.739	0.399	24.2
**Tel**	80.0	4.03	0.740	0.370	23.5
**TEs**	54.2	4.50	0.646	0.331	17.2
**TS**	66.1	3.13	0.234	0.355	24.3
**VD**	73.7	5.09	0.478	0.290	17.5

Native structure recognition performance comparison among scoring functions. All reported measures are averages over the 77 decoy sets in the final testing group. Lower scores are better for Rank_nat _and RMSD_best_. Higher ones are better for Z_nat_, CC_nat _and FE_nat_. LoCo outperforms all other functions except DFMAC in every measure. All metrics are defined in *Performance measures *at the end of **Methods**.

Decoy discrimination is shown in Table 4. Again, LoCo and DFMAC were the top two functions in most measures. LoCo had the best R_B10_, RMSD_decoy _and log(P_B1_). It was slightly lower than DFMAC in Z_decoy_, CC_decoy _and FE_decoy_, and it was slightly higher than ProSa 2003 in log(P_B10_).

**Table 4:** Function comparison: decoy discrimination

	R_B1_	R_B10_	RMSD_decoy_	Z_decoy_	CC_decoy_	FE_decoy _(%)	log(P_B1_)	log(P_B10_)
**LoCo**	154.8	5.6	3.51	0.938	0.505	31.4	-0.864	-1.640
**DFMAC**	108.9	13.8	3.64	1.024	0.533	31.6	-0.825	-1.586
**RF_CB_SRS_OD**	172.8	52.5	4.11	0.914	0.457	28.4	-0.761	-1.524
**ProSa 2003**	118.2	24.8	3.82	0.931	0.493	32.3	-0.755	-1.650
**Four-body**	124.8	36.8	5.01	0.539	0.328	19.6	-0.488	-1.317
**General-four-body**	186.3	52.2	4.97	0.436	0.312	17.1	-0.482	-1.241
**Short-range**	192.4	36.6	5.27	0.377	0.267	14.9	-0.536	-1.249
**BFKV**	139.4	32.3	3.98	0.673	0.398	25.3	-0.671	-1.412
**BT**	175.4	39.0	4.32	0.636	0.391	24.3	-0.558	-1.406
**GKS**	164.6	71.3	5.57	0.187	0.231	13.0	-0.400	-1.166
**HLPL**	161.3	31.7	3.86	0.725	0.396	27.3	-0.650	-1.434
**MJ1**	183.1	34.9	4.54	0.596	0.336	22.5	-0.594	-1.302
**MJ2h**	149.5	28.4	3.93	0.695	0.407	25.1	-0.640	-1.361
**MJ3**	172.4	25.3	4.92	0.270	0.241	15.8	-0.476	-1.241
**MJ3h**	152.2	26.5	4.00	0.641	0.416	25.7	-0.615	-1.417
**MJPL**	129.5	29.0	4.15	0.693	0.375	25.6	-0.650	-1.363
**MS**	191.1	67.1	5.09	0.217	0.236	13.4	-0.496	-1.155
**MSBM**	140.1	65.4	5.77	-0.001	0.165	7.6	-0.359	-1.043
**Qa**	176.9	32.0	5.02	0.357	0.286	17.2	-0.480	-1.323
**Qm**	189.6	53.8	5.02	0.356	0.268	16.9	-0.479	-1.224
**Qp**	163.9	31.4	3.81	0.721	0.377	25.0	-0.622	-1.381
**RO**	231.3	44.3	5.83	0.197	0.240	15.5	-0.396	-1.194
**SKJG**	146.4	41.1	4.45	0.504	0.362	20.4	-0.586	-1.309
**SKOa**	90.7	37.1	4.46	0.469	0.352	20.8	-0.593	-1.328
**SKOb**	144.3	27.9	4.34	0.573	0.363	21.8	-0.587	-1.395
**TD**	156.4	30.6	4.17	0.724	0.416	26.3	-0.604	-1.418
**Tel**	141.3	64.4	4.26	0.540	0.363	22.0	-0.569	-1.402
**TEs**	163.3	62.1	4.74	0.523	0.338	18.5	-0.522	-1.237
**TS**	125.5	28.7	4.04	0.697	0.381	26.3	-0.656	-1.358
**VD**	149.8	48.4	5.22	0.504	0.292	17.3	-0.435	-1.284

Comparison of decoy discrimination performance among all tested functions is shown. All reported measures are averages over the 77 decoy sets in the final testing group. Lower scores are better for R_B1_, R_B10_, RMSD_decoy_, log(P_B1_) and log(P_B10_). Higher scores are better for Z_decoy_, CC_decoy _and FE_decoy_. LoCo outperforms all other functions in R_B10_, RMSD_decoy_, and log(P_B1_). It is slightly higher than ProSa 2003 in log(P_B10_) and slightly lower than ProSa 2003 in FE_decoy_. Z_decoy_, CC_decoy _and FE_decoy _for LoCo are all slightly lower than for DFMAC. LoCo outperforms the remaining 27 functions in every measure except R_B1_. All metrics are defined in *Performance measures *at the end of **Methods**.

### All-atom function comparison

To get a sense of how our main chain-only function compares to available all-atom functions, we tested four widely-used potentials that work with all heavy atom coordinates on the same final testing group of decoys we have used throughout. The potentials chosen were RAPDF [53], dDFIRE [54,55], DOPE [50] and RF_HA_SRS [45]. These functions require that all side chain atoms be included and their positions determined in every structure to be scored.

LoCo performance compared to these four potentials for native structure recognition is shown in Table 5, while performance for decoy discrimination is shown in Table 6. The performance of LoCo was quite comparable to these higher-resolution functions. LoCo outperformed all four in Rank_nat_, R_B10 _and log(P_B10_). It placed no worse than third (of five) in every performance metric except R_B1_.

**Table 5:** LoCo vs. all-atom potentials: native recognition

	Rank_nat_	RMSD_best_	Z_nat_	CC_nat_	FE_nat _(%)
**LoCo**	13.4	1.62	1.805	0.519	36.6
**RAPDF**	30.2	2.54	1.367	0.474	33.2
**dDFIRE**	21.2	1.89	2.019	0.556	37.3
**DOPE**	37.5	2.69	1.525	0.482	34.5
**RF_HA_SRS**	18.6	1.59	2.055	0.526	39.7

LoCo native recognition performance is compared to that of four widely-used all-atom potentials. All reported measures are averages over the 77 decoy sets in the final testing group. LoCo performance is comparable to the others, placing 1^st^, 2^nd^, 3^rd^, 3^rd ^and 3^rd ^in Rank_nat_, RMSD_best_, Z_nat_, CC_nat _and FE_nat_, respectively. Taking the sum of all rankings among these five potentials, LoCo places 3^rd ^overall. All metrics are defined in *Performance measures *at the end of **Methods**.

**Table 6:** LoCo vs. all-atom potentials: decoy discrimination

	R_B1_	R_B10_	RMSD_decoy_	Z_decoy_	CC_decoy_	FE_decoy _(%)	log(P_B1_)	log(P_B10_)
**LoCo**	154.8	5.6	3.51	0.938	0.505	31.4	-0.864	-1.640
**RAPDF**	152.2	27.7	4.02	0.878	0.479	30.8	-0.818	-1.604
**dDFIRE**	136.5	18.9	3.75	1.014	0.536	33.3	-0.896	-1.592
**DOPE**	97.4	52.0	4.21	0.764	0.466	25.9	-0.717	-1.409
**RF_HA_SRS**	122.5	56.9	3.45	0.896	0.493	33.1	-0.881	-1.526

Decoy discrimination performance for LoCo is compared to that of four widely-used all-atom potentials. All reported measures are averages over the 77 decoy sets in the final testing group. LoCo outperforms all others at R_B10 _and log(P_B10_). LoCo is beaten by all four at R_B1_. LoCo places 2^nd ^among all potentials at RMSD_decoy_, Z_decoy _and CC_decoy_. It places 3^rd ^in FE_decoy _and log(P_B1_). When the sum of all rankings among these five potentials is considered, LoCo places 2^nd ^overall. All metrics are defined in *Performance measures *at the end of **Methods**.

### Speed

LoCo is extremely fast, particularly compared to other functions that are based on explicit distance calculations and table lookups. Scoring for LoCo was carried out on an Apple iMac with a 2.4 GHz Intel Core 2 Duo processor with 4 GB of memory. The function was written in C++ and compiled using GNU g++ 4.2.

The average total processing time for a single structure in the final testing group was 2.6 milliseconds. This time includes reading the structure from the hard disk drive, loading it into the program, determining all relevant interactions, scoring the structure and clearing it from memory. The average time for interaction determination and scoring only was 0.47 ms. The numbers of residues per structure in the final testing group varied from 31 to 274, so the standard deviations for total processing time and interaction determination and scoring time were relatively large: 1.4 ms (54%) and 0.32 ms (68%), respectively.

On a "per residue" basis the times were more consistent. The average total processing time per residue was 0.032 ms with a standard deviation of 0.0037 ms (12%). The average interaction determination and scoring time per residue was 0.0054 ms with a standard deviation of 0.0011 ms (20%).

The time taken by LoCo to score the entire final testing set of 39,611 structures, including reading the scoring databases, input structures, and writing the output score files is ~4 minutes. We were unable to determine the amount of time needed by DFMAC or any of the server functions to score the entire final testing group, but ProSa 2003 takes ~121 minutes and RF_CB_SRS_OD takes 11 minutes.

In contrast an all atom scoring function, RAPDF [53], pioneered by our group takes several seconds on average to score a structure from scratch as described above, and about one second for interaction determination and scoring only. The backbone only version of this function is about ten fold faster but still takes about 100 ms per structure. Thus a very rough comparison indicates that LoCo is approximately two orders of magnitude faster compared to traditional distance bin based potentials of mean force.

### Statistical significance

**Figure 3:**
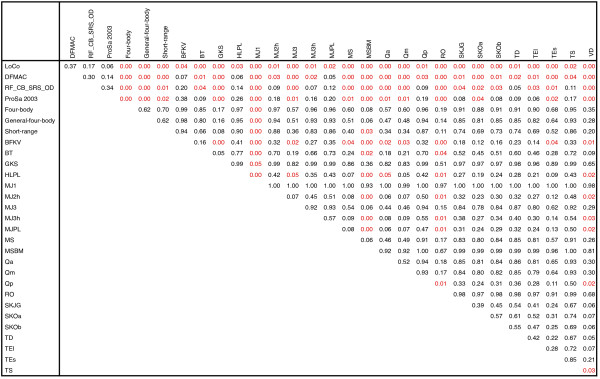
**Statistical significance of differences in rank distributions**. C_α _RMSD rank distributions for the best-scoring non-native structures for all functions are compared. P-values show the likelihood that better rank distributions for the function on the left are the result of chance. P-values less than 0.05 have been colored in red, showing statistically significant differences in these distributions. These ranks are among decoy structures only. The null hypothesis of this one-tailed Wilcoxon test is that neither distribution is lower than the other. The alternative hypothesis is that functions on the left achieved lower ranks for their best-scoring decoys than functions along the top.

To assess the statistical significance of differences between potentials in the distribution of ranks, we performed pairwise one-tailed Wilcoxon tests on all tested functions. We used R_B1_, the C_α _RMSD rank (among decoys only) of the best-scoring decoy structure as our tested distribution. We felt that this was the closest suitable metric to RMSD_decoy_, the one on which the LoCo potential was parameterized. We also believe that it best encompasses our primary goal of picking out the nearest native decoy structures. Results of this test are in Figure 3.

Our null hypothesis was that neither function performed better in the the distributions of these ranks, and our alternative hypothesis was that the function in the leftmost column of Figure 3 had a distribution of ranks that were lower than that of the function in the column across the top, showing that the functions in the left column performed better. The Wilcoxon test was used because the rank distributions being compared are far from normal.

The large number of red values in the top four rows (p-value < 0.05) show that LoCo, DFMAC, RF_CB_SRS_OD and ProSa 2003 have statistically significant differences in rank distribution from most of the other 26 functions, based on the hypothesis that their distributions are lower. These p-values represent the likelihood that the better rankings for the functions on the left could have come about by chance. The ranks for LoCo were better than all other functions, since all p-values were < 0.5. However, these rank distributions vs. DFMAC, RF_CB_SRS_OD and ProSa 2003 were not below the statistical significance threshold of 0.05.

## Discussion

### Relative importance of performance measures

The primary goal of a main chain-only scoring function is to identify proposed main chain conformations that are reasonably likely to be close enough to the native structure to be kept for more detailed evaluation. A large number of possible main chains are typically tried, and the likelihood that any of them will be exactly the same as the native is very small. For this reason, we believe that good performance in decoy discrimination is more important than good performance in native structure recognition.

We also consider R_B1_, R_B10_, RMSD_decoy_, log(P_B1_) and log(P_B10_) to be more important to the goal of selecting a relatively small number of near native decoys than Z_decoy_, CC_decoy _and FE_decoy_. R_B1 _and R_B10 _inform whether or not the very best-scoring decoys are among the very closest to the native. RMSD_decoy _tells how close to correct the best-scoring decoy is. Log(P_B1_) and log(P_B10_) gives us measures of how meaningful the R_B1 _and R_B10 _values are.

Other metrics, while still valuable, are less directly related to the goal of finding near native structures. Z_decoy _measures how far from the mean score our best decoy is, but what matters most is whether we can identify it. CC_decoy _reveals the correspondence between score and RMSD across the entire set, but this correspondence is of little importance for poor decoys that will be rejected. FE_decoy _assesses performance with the top 10% of decoys, but at the initial main chain evaluation stage, we are likely to be keeping far fewer than 10% of the main chain conformations we examine.

### LoCo vs. DFMAC

LoCo outperformed all other functions in R_B10_, RMSD_decoy_, and log(P_B1_), three of the five measures most important for finding near native decoys. It was only slightly higher than ProSa 2003 at log(P_B10_). Its R_B1 _was higher than many of the other functions, but since any initial main chain search will keep more than one structure for further evaluation, LoCo's lowest R_B10 _should be considered more relevant. At native structure recognition, LoCo's performance was just behind that of DFMAC in all categories, although it was still substantially better than the remaining 28 functions.

While we consider the performance of LoCo and DFMAC to be roughly comparable, we believe that LoCo has clear practical advantages over DFMAC. DFMAC is a weighted composite of six separate functions that require the creation of pseudo-N, -O, -H and - C_β _atoms for every residue as well as the calculation of at least five angles between vectors for every residue-residue interaction and three dihedral angles for every residue. These angle calculations are computationally expensive and must be repeated for every new main chain conformation.

LoCo, on the other hand, was designed to be extremely fast. Every residue-residue interaction requires only a single lookup from the potential database. The initial C_α_→ C_α _vector between any two residues being scored undergoes a single matrix rotation into the local coordinate system of the observing residue, where it is then binned and the score for the interaction is looked up. The initial generation of the rotation matrix that defines the local coordinate system does require several computationally expensive square root and trigonometric operations per residue, but all translations and rotations of the main chain after that require only simple arithmetic floating-point operations, including rotating the coordinate system.

DFMAC was also finely tuned to its training set, with sixteen independent parameters and five weights optimized to give the best possible performance. These training procedures were carried out with rigor to ensure that no structure was scored using parameters that had been trained on it, but all decoy sets used for training had been generated using the same methods employed to create the decoy sets in the testing group. 15 of the 77 decoy sets in the final testing group had as their native structures proteins that appeared in the training group as part of decoy sets generated by alternate methods. In 12 of those 15 sets the native was correctly identified by DFMAC. It is unclear to us that the values of those parameters and weights used by DFMAC will be optimal for the prediction of protein structures more generally.

LoCo, on the other hand, is largely insensitive to changes in its parameters. We compared the best, worst and average values for each individual performance measure across all 84 LoCo parameter sets with the performance of DFMAC, ProSa 2003, and RF_CB_SRS_OD. We also compared them with the best, worst and average values for all 26 functions from the Jernigan Lab server.

Tables 7 and 8 indicate that the differences in performance between LoCo parameter sets were not large. For native recognition (Table 7), the average value for LoCo across all 84 parameter sets in any of the five performance measures were still better than for any potential other than DFMAC. The worst LoCo value was better than the best value for any of the Jernigan server potentials in 4 out of 5 cases, and the worst LoCo CC_nat _of 0.403 was only 0.007 lower than the best Jernigan server CC_nat _of 0.410.

**Table 7:** LoCo variation: native recognition

	Rank_nat_	RMSD_best_	Z_nat_	CC_nat_	FE_nat _(%)
**LoCo BEST**	12.0	1.51	1.870	0.529	38.4
**LoCo WORST**	17.5	3.09	1.445	0.403	30.0
**LoCo AVERAGE**	13.9	2.36	1.659	0.496	34.5
**LoCo CHOSEN**	13.4	1.62	1.805	0.519	36.6
**DFMAC**	6.7	1.17	2.630	0.562	38.3
**RF_CB_SRS_OD**	19.3	2.68	1.508	0.464	31.3
**ProSa 2003**	44.0	2.39	1.288	0.491	33.8
**Server BEST**	28.5	3.12	0.797	0.410	26.3
**Server WORST**	248.3	5.77	0.014	0.159	7.5
**Server AVERAGE**	63.3	4.27	0.521	0.324	19.9

Best, worst and average performance for LoCo across all 84 parameter sets tested is compared with the chosen LoCo parameter set, the three best-performing of the other potentials, and the best, worst and average performance of all 26 remaining potentials from the Jernigan Lab server. All best, worst, and average values are for each individual performance measure; no single set contained all those values. All reported measures are averages over the 77 decoy sets in the final testing group. All metrics are defined in Performance measures at the end of **Methods**. In summary, lower scores are better for Rank_nat _and RMSD_best_. Higher ones are better for Z_nat_, CC_nat _and FE_nat_. The average performance across all 84 versions of LoCo surpassed that every other function except DFMAC. Even at its worst, performance for LoCo exceeded that of all Jernigan server functions for every measure except CC_nat_.

**Table 8:** LoCo variation: decoy discrimination

	R_B1_	R_B10_	RMSD_decoy_	Z_decoy_	CC_decoy_	FE_decoy _(%)	log(P_B1_)	log(P_B10_)
**LoCo BEST**	25.5	5.6	3.01	1.005	0.517	33.0	-0.982	-1.654
**LoCo WORST**	175.0	53.0	4.09	0.748	0.374	24.0	-0.665	-1.432
**LoCo AVERAGE**	112.5	26.5	3.68	0.909	0.481	29.9	-0.778	-1.562
**LoCo CHOSEN**	154.8	5.6	3.51	0.938	0.505	31.4	-0.864	-1.640
**DFMAC**	108.9	13.8	3.64	1.024	0.533	31.6	-0.825	-1.586
**RF_CB_SRS_OD**	172.8	52.5	4.11	0.914	0.457	28.4	-0.761	-1.524
**ProSa 2003**	118.2	24.8	3.82	0.931	0.493	32.3	-0.755	-1.650
**Server BEST**	90.7	25.3	3.81	0.725	0.416	27.3	-0.671	-1.434
**Server WORST**	231.3	71.3	5.83	-0.001	0.165	7.6	-0.359	-1.043
**Server AVERAGE**	159.3	41.5	4.64	0.494	0.328	20.2	-0.544	-1.306

Best, worst and average performance for LoCo across all 84 parameter sets tested is compared with the chosen LoCo parameter set, the three best-performing of the other potentials, and the best, worst and average performance of all 26 remaining potentials from the Jernigan Lab server. All best, worst, and average values are for each individual performance measure; no single set contained all those values. All reported measures are averages over the 77 decoy sets in the final testing group. Lower scores are better for R_B1_, R_B10_, RMSD_decoy_, log(P_B1_) and log(P_B10_). Higher scores are better for Z_decoy_, CC_decoy _and FE_decoy_. The average performance for LoCo among all 84 parameter sets exceeds all other functions except DFMAC in RMSD_decoy _and log(P_B1_). The LoCo average betters all other functions except DFMAC and ProSa 2003 in log(PB10). All metrics are defined in *Performance measures *at the end of **Methods**.

For decoy discrimination (Table 8), the best value for LoCo across all parameter sets was better than any other function for all performance measures, with the exception of Z_decoy _and CC_decoy _for DFMAC. The average value for LoCo across all sets was better than the best values from the Jernigan server potentials for 6 out of 8 measures. It was also better than RF_CB_SRS_OD for 7 of 8 measures, with a slightly worse Z_decoy_.

### Omega angles

The DFMAC function includes an ω angle term. The ω is the main chain dihedral angle between the C_α_→C vector of one residue and the C_α_→N vector of the following residue. In an experimentally determined structure these angles are typically clustered around 180° because of the partially double-bonded character of most C_α_→C→N→ C_α _groups. There are usually a few places within any main chain where the planarity of this system is broken to make energetically favorable interactions elsewhere, but the great majority of native ω angles are within 15° to either side of a planar 180° separation.

It is unlikely that any initial main chain conformational search would include variations of the ω angle, since that would introduce unnecessary degrees of freedom to achieve only slight differences in the overall structure. An ω angle function can, however, be quite effective at distinguishing native main chain geometry from that of computer-generated decoys. This is because these variations are often more characteristic of the method used to generate the decoys than of structural correctness.

To demonstrate this point, we created a very simple ω angle discrimination function. It calculates the standard deviation of all individual ω angles for any main chain that are within 15° of 180° apart. The score for each main chain is the magnitude of the difference (in degrees) between its own standard deviation and the mean of all the standard deviations in the decoy set.

For purposes of illustration only, we have included this function in Tables 9 and 10 and have compared it to the performance of LoCo and of DFMAC both with and without the ω angle score component. For native recognition (Table 9), our ω-only function is able to recognize native structures (Rank_nat_) very nearly as well as DFMAC without an ω angle component. The Z_nat _of the ω-only function is more than twice as great as either version of DFMAC. Its RMSD_best _is better than every function tested except LoCo, DFMAC and ProSa 2003, and it is within 0.01Å of ProSa 2003.

**Table 9:** Omega angles and native recognition

	Rank_nat_	RMSD_best_	Z_nat_	CC_nat_	FE_nat _(%)
**LoCo**	13.4	1.62	1.805	0.519	36.6
**OMEGAS ONLY**	12.1	2.40	5.640	0.198	18.6
**DFMAC WITH OMEGAS**	6.7	1.17	2.630	0.562	38.3
**DFMAC WITHOUT OMEGAS**	11.9	1.04	2.582	0.558	39.0

Native recognition performance comparison among LoCo, our ω-only function and DFMAC both with and without its ω component is shown. All reported measures are averages over the 77 decoy sets in the final testing group. Lower scores are better for Rank_nat _and RMSD_best_. Higher ones are better for Z_nat_, CC_nat _and FE_nat_. The ω-only function is able to pick out native structures quite well, but when it fails, its choices are essentially random. In the two measures for which the ω-only function does poorly (CC_nat _and FE_nat_), DFMAC performance improves when its ω component is removed. All metrics are defined in *Performance measures *at the end of **Methods**.

**Table 10:** Omega angles and decoy discrimination

	R_B1_	R_B10_	RMSD_decoy_	Z_decoy_	CC_decoy_	FE_decoy _(%)	logP_B1_	logP_B10_
**LoCo**	154.8	5.6	3.51	0.938	0.505	31.4	-0.864	-1.640
**OMEGAS ONLY**	171.1	47.9	6.46	0.100	0.166	9.1	-0.361	-1.226
**DFMAC WITH OMEGAS**	108.9	13.8	3.64	1.024	0.533	31.6	-0.825	-1.586
**DFMAC WITHOUT OMEGAS**	106.1	12.6	3.61	1.021	0.533	32.1	-0.830	-1.600

Comparison of decoy discrimination performance comparison among LoCo, our ω-only function and DFMAC, both with and without its ω component, is shown. All reported measures are averages over the 77 decoy sets in the final testing group. Lower scores are better for R_B1_, R_B10_, RMSD_decoy_, log(P_B1_) and log(P_B10_). Higher scores are better for Z_decoy_, CC_decoy _and FE_decoy_. Performance for our ω-only function is approximately the same as if its choices had been made at random. With the exception of CCdecoy (which stays the same) DFMAC performance improves across the board with the ω component removed. All metrics are defined in *Performance measures *at the end of **Methods**.

For DFMAC, Z_nat _improves noticeably and Rank_nat _improves significantly with the inclusion of the ω angle component while RMSD_best _and FE_nat _decline slightly. This mirrors the very good scores of the ω-only function for Rank_nat _and Z_nat _and its relatively poor performance at FE_nat_. The slight decline in RMSD_best _for DFMAC when the ω angle component is included must be considered an artifact of the tenfold cross-validation used when weighting the various DFMAC components. This is because that performance measure was the one being optimized and because the ω angle component was assigned a positive weight.

With native structures removed (Table 10), the decoys selected by our ω-only function are effectively random. DFMAC performance improves slightly across the board without the ω component. This suggests that using ω angles improves some performance measures of native structure recognition but degrades decoy discrimination.

### LoCo Applications

LoCo potentials combine speed, accuracy and ease of implementation. They should be of use in a variety of structure prediction tasks, including both template based (homology) and template free (*ab initio*) modeling. We anticipate that they will be accurate enough to allow for improved main chain-only refinement of template based models before they are treated at the all-atom level.

We expect that our potentials will be useful for protein design applications as well. Currently successful sequence search algorithms must evaluate structures at an all-atom level [56,57]. This means that they cannot fully sample the sequence space but must rely on more restricted search techniques, such as a Monte Carlo method [58]. A sufficiently accurate main chain-only potential function should allow the entire sequence space to be searched, treating design as a combinatorial optimization problem, much like choosing side chain conformations.

With its speed and accuracy, LoCo is a good candidate for such an application. The stablest possible sequence for a given main chain is the global minimum energy conformation (GMEC). A low-resolution function like LoCo would be unlikely to arrive at the GMEC, but it would not need to. The LoCo-designed sequence would only need to be stable enough for the desired application. Even if the LoCo-designed sequence was not stable enough to be used, it should provide a good starting point for further refinement using all-atom methods.

### Future directions

While these potentials have been developed for and with complete polypeptide chains, there may well be value in developing individual potentials for secondary structure elements and loops. Such potentials may be able to aid in the recognition of helices and sheets within sequences for which no homolog is known, and loop-specific functions may aid in faster and more accurate modeling of the most challenging aspect of protein structure prediction. As noted above, we hope that LoCo will allow for a broader search of protein sequence space in design applications.

The idea behind LoCo scoring should also work for low-resolution screening of docked protein-protein complexes. Currently, initial-stage docking programs are dominated by grid-based algorithms [59] that rely on fast-fourier-transforms (FFTs) to provide the speed necessary to sample all possible docked conformations in a reasonable amount of time, which may be improved by a LoCo type potential for docking.

## Conclusions

We present a novel scoring function, "LoCo," for evaluating protein main chain conformations. Our method considers relative positioning in all three dimensions and examines every interaction from the perspective of both partners, in contrast with every other function it was tested against. A number of recently-developed potentials have achieved improved performance over more traditional contact potentials by considering the relative orientations of two interacting residues.

LoCo provides an unprecedented combination of speed and accuracy. Once an interaction has been characterized by the identities of the participating residues and their relative positions, a single lookup gives the score for that particular interaction. This function has many potential uses in the field of protein structure prediction, and since a local coordinate system can be generated for any chiral group of atoms, there are many possible ways the fundamental concept could be applied.

## Competing interests

The authors declare that they have no competing interests.

## Authors' contributions

SM conceived the function, wrote the software, carried out all function training and decoy testing and drafted the manuscript. RS supervised the research, edited the manuscript, and provided intellectual mentorship. All authors read and approved the final manuscript.
